# Prevalence of Obstructive Sleep Apnea Is Increased in Patients With Inflammatory Bowel Disease: A Large, Multi-Network Study

**DOI:** 10.1093/crocol/otac026

**Published:** 2022-07-06

**Authors:** Kyle Hoffman, Emad Mansoor, Muhammad Siyab Panhwar, Miguel Regueiro, Gregory Cooper, Taha Qazi

**Affiliations:** Case Western Reserve University/University Hospitals Cleveland Medical Center, Department of Medicine, Cleveland, Ohio, USA; Cleveland Louis Stokes VA Medical Center, Division of Gastroenterology and Liver Disease, Cleveland, Ohio, USA; Department of Medicine, Tulane University Medical Center, Section of Cardiology, New Orleans, Louisiana, USA; Cleveland Clinic Foundation, Department of Gastroenterology, Cleveland, Ohio, USA; Case Western Reserve University/University Hospitals Cleveland Medical Center, Division of Gastroenterology, Cleveland, Ohio, USA; Cleveland Clinic Foundation, Department of Gastroenterology, Cleveland, Ohio, USA

**Keywords:** inflammatory bowel disease, obstructive sleep apnea, Crohn’s disease, ulcerative colitis

## Abstract

**Background:**

Crohn’s disease (CD) and ulcerative colitis (UC) involve an inflammatory state where sleep dysregulation is common. Little is known about implications, if any, of inflammatory bowel disease (IBD) on the development of obstructive sleep apnea (OSA). This study aims to investigate if IBD patients are at higher risk for OSA.

**Methods:**

This retrospective multivariate analysis utilized a commercial database named Explorys (IBM Watson). We identified patients from 1/2015 to 1/2020 with UC and CD. Cohorts of these patients with and without OSA were then created and prevalence values were obtained. A multivariate analysis was used to correct for several potential confounding variables.

**Results:**

The overall prevalence of OSA was 7.8% in UC and 7.2% in CD, as compared with a prevalence of 4.3% in non-IBD patients (odds ratio [OR] for UC: 1.9 [95% CI 1.86–1.94, *P* < .0001], OR for CD: 1.72 [95% CI 1.69–1.76, *P* < .0001]). In multivariate analysis, age above 65, Caucasian race, male sex, obesity, smoking, hypertension, and diabetes were all independent risk factors for the development of OSA, with obesity being the most significant. After controlling for the listed variables in the multivariate analysis, IBD was an independent risk factor associated with OSA (OR 1.46, 95% CI 1.43–1.48).

**Conclusions:**

In this large population-based study, IBD was independently associated with increased prevalence of OSA. This has implications for screening for OSA in IBD, as well as management of other risk factors for OSA in IBD.

## Introduction

The pathogenesis of obstructive sleep apnea (OSA) is multifactorial and heterogeneous. However, a factor in the pathophysiology is enlargement of the soft tissues of the upper airway.^1^ This can occur as the result of inflammation and edema, either locally, as the result of trauma caused by vibration during snoring, or systemically.^1^

The inflammatory bowel diseases (IBD), comprised of Crohn’s disease (CD) and ulcerative colitis (UC), are present in an estimated 1.5 million people across North America and 2.5 million people across Europe.^2^ Both CD and UC are mediated by inflammatory cytokines through specific types of T cells that are activated, but the cytokine profiles differ between the 2 diseases.^3^ Through similar mechanisms, this inflammatory state affects both the gastrointestinal tract and extraintestinal sites, such as the kidneys, skin, and liver.^4^ Previous studies have demonstrated an association between IBD and persistent airway inflammation, which, can manifest as tracheal stenosis and bronchiectasis.^4^

IBD leads to higher risk for cardiovascular disease, with a recent study showing a link between IBD and acute myocardial infarction.^5^ OSA also results in a proinflammatory state and is associated with coronary heart disease, heart failure, and arrhythmias.^6^ Given that both IBD and OSA portend a high risk for morbidity and mortality and a potential link between the 2 disease processes has not been previously described, this study aims to investigate whether or not IBD patients are at higher risk of OSA.

## Methods

### Study Design

This retrospective analysis utilized a large electronic medical record (EMR)-based database named Explorys (IBM Watson). This database aggregates inpatient and outpatient EMR data from 26 major integrated healthcare systems that span across all 50 states. Data have been collected from 1999 onwards, resulting in the collection of more than 63 million unique records.^7^ It has previously been validated for use in several different medical subspecialties.^8–10^

Deidentified data from the electronic health records, billing records, and laboratory records of each participating institution are standardized, normalized, and then stored in a cloud-based database utilizing standardized medical ontologies, such as the Systematized Nomenclature of Medicine Clinical Terms (SNOMED-CT) for medical diagnoses and procedures, RxNorm for medications, and logical observation identifier names and codes (LOINC) for laboratory values. *International Classification of Disease* diagnosis codes are mapped into the SNOMED-CT hierarchy, as well. This database then allows users to create cohorts of patients based on the presence or absence of SNOMED-CT diagnoses with further subdivision of cohorts based on demographic information, as well as comorbid conditions. Further information regarding the Explorys database is previously described elsewhere.^8^

Explorys is Health Insurance Portability and Accountability Act (HIPAA) compliant and is considered exempt from the University Hospitals Institutional Review Board review process. However, Explorys rounds population counts to the nearest 10, treating all values between 0 and 10 as equivalent, with all values between 1 and 10 approximated to be 5.

### Patient Selection

Utilizing the Explorys search tool, we identified all nondeceased patients with active records within the last 5 years (January 2015–January 2020). We then identified all patients with UC and CD utilizing the search terms “ulcerative colitis” and “Crohn’s disease,” respectively. Patients with diagnoses of both UC and CD were excluded. Patients with OSA were identified using the SNOMED-CT diagnosis of “obstructive sleep apnea syndrome.” Cohorts of patients with UC or CD with and without OSA were created. A control cohort of patients without UC or CD with and without OSA was also formed. We used the “index event” feature on Explorys to allow us to only identify patients who were diagnosed with OSA after a diagnosis of UC or CD.

### Covariates

Within the cohorts, patient demographic information was collected including age, sex, and race (Caucasian, African-American, Asian, Hispanic). The major risk factors for OSA are obesity, smoking, family history, and craniofacial abnormalities.^11–13^ Using the SNOMED-CT terms of “tobacco user” and “obesity,” as well as “benign hypertension” and “diabetes mellitus,” data for some of the major risk factors and comorbidities for this disease process were collected. Other major risk factors, such as craniofacial abnormalities and family history, were unable to be assessed using Explorys.

### Statistical Analysis

Subdivided by each age group (18–65, 65+), as well as sex, race, and presence or absence of the above comorbid conditions, the prevalence of UC and CD was obtained, as was the number of patients without IBD.

The prevalence of OSA in UC was determined by dividing the total number of patients who developed OSA after a diagnosis of UC by the total number of patients with UC. Similarly, the prevalence of OSA in CD and in non-IBD patients was calculated.

A multivariate model was created using the “fast search” tool in Explorys to identify those active within the last 3 years, a new cohort independent of that previously studied, and adjusting for the following potential confounding variables: age, race, sex, obesity, smoking, benign hypertension, and diabetes mellitus. With OSA as the dependent variable, odds ratios (ORs) were calculated, along with their respective 95% CIs. The associated *P* values were also calculated, with *P* < .05 being considered statistically significant.

## Results

Among active patients in the database from January 2015 to January 2020, there were 117 510 with a diagnosis of UC, 145 590 with a diagnosis of CD, and 40 707 320 without either diagnosis. Table 1 shows baseline characteristics of the studied population, including age, race, sex, and comorbid conditions.

**Table 1.** Baseline characteristics of patients with and without inflammatory bowel disease.

	UC patients (proportion)	CD patients (proportion)	Non-IBD patients (proportion)	*P* (UC vs CD)	*P* (UC vs non-IBD)	*P* (CD vs non-IBD)
18–65	71 970 (0.61)	101 640 (0.70)	24 386 110 (0.60)	<.0001	<.0001	<.0001
>65	44 380 (0.38)	40 750 (0.28)	8 906 160 (0.22)	<.0001	<.0001	<.0001
Caucasian	94 580 (0.80)	114 410 (0.79)	24 230 180 (0.60)	<.0001	<.0001	<.0001
Non-Caucasian (excluding refuse to classify)	28 260 (0.24)	36 220 (0.25)	11 827 670 (0.29)	<.0001	<.0001	<.0001
Male	48 710 (0.41)	58 570 (0.40)	17 845 460 (0.44)	<.0001	<.0001	<.0001
Female	66 180 (0.56)	83 910 (0.58)	22 103 950 (0.54)	<.0001	<.0001	<.0001
Obesity	22 550 (0.19)	26 460 (0.18)	4 193 630 (0.10)	<.0001	<.0001	<.0001
Smoking (“tobacco user”)	22 520 (0.19)	38 960 (0.27)	5 274 270 (0.13)	<.0001	<.0001	<.0001
HTN (“essential hypertension”)	54 080 (0.46)	59 340 (0.41)	9 540 050 (0.23)	<.0001	<.0001	<.0001
DMII (“diabetes mellitus, type 2”)	21 820 (0.19)	22 970 (0.16)	3 729 090 (0.09)	<.0001	<.0001	<.0001

Abbreviations: CD, Crohn’s disease; IBD, inflammatory bowel disease; UC, ulcerative colitis.

**Figure 1.**
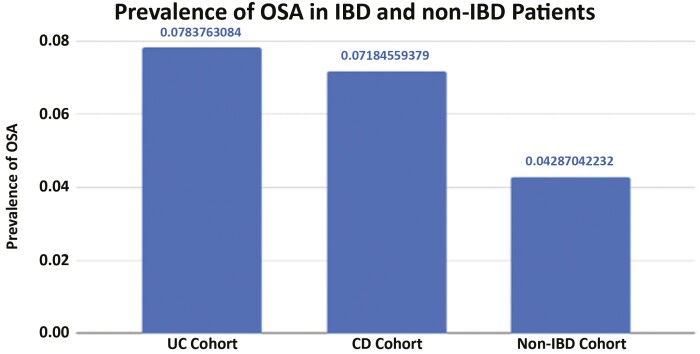
Prevalence of obstructive sleep apnea in patients with and without inflammatory bowel disease. OSA was present in 9210 patients who had ulcerative colitis, a significantly higher prevalence than without IBD (7.8% vs 4.3%, OR 1.90, 95% CI 1.86–1.94, *P* < 0.0001). OSA was present in 10 460 patients, also significantly higher than those without IBD (7.2% vs 4.3%, OR 1.72, 95% CI 1.69–1.76, *P* < 0.0001). Abbreviations: CD, Crohn’s disease; IBD, inflammatory bowel disease; OR, odds ratio; OSA, obstructive sleep apnea; UC, ulcerative colitis.

The results of the unadjusted analysis are presented in Figure 1. OSA was present in 9210 patients who had UC, a significantly higher prevalence than those without IBD (7.8% vs 4.3%, OR 1.90, 95% CI 1.86–1.94, *P* < .0001). In CD patients, OSA was present in 10 460 patients, also significantly higher than those without IBD (7.2% vs 4.3%, OR 1.72, 95% CI 1.69–1.76, *P* < .0001). In addition, Table 2 shows the results of the unadjusted analysis when further subcategorized by age, race, and comorbidity. There is significant variability in the effects of IBD on the rates of OSA among the subgroups, but ORs in each subcategory demonstrated a significant increase in risk for OSA for both Crohn’s and UC, as compared with non-IBD patients.

**Table 2.** Prevalence of obstructive sleep apnea with and without inflammatory bowel disease by age, race, and risk factor.

	Age 18–65	Age 65+	Caucasian	Non-Caucasian	Obesity	Smoking	Hypertension	Diabetes
OSA in UC (*N* = 9210)	4330 (0.47); OR 1.55 (95% CI 1.50–1.60)	4910 (0.53); OR 1.41 (95% CI 1.37–1.45)	7790 (0.85); OR 1.55 (95% CI 1.51–1.58)	2470 (0.27); OR 2.01 (95% CI 1.93–2.10)	5240 (0.57); OR 1.05 (95% CI 1.02–1.09)	2430 (0.26); OR 1.41 (95% CI 1.35–1.47)	7280 (0.79); OR 1.06 (95% CI 1.03–1.08)	3920 (0.43); OR 1.07 (95% CI 1.03–1.11)
OSA in CD (*N* = 10 460)	5840 (0.56); OR 1.47 (95% CI 1.44–1.51)	4600 (0.44); OR 1.44 (95% CI 1.40–1.48)	8700 (0.83); OR 1.42 (95% CI 1.39–1.45)	3000 (0.29); OR 1.90 (95% CI 1.83–1.97)	6190 (0.59); OR 1.06 (95% CI 1.03–1.09)	3520 (0.34); OR 1.16 (95% CI 1.12–1.20)	8140 (0.78); OR 1.08 (95% CI 1.06–1.11)	4460 (0.43); OR 1.18 (95% CI 1.14–1.22)
OSA in non-IBD (*N* = 1 745 140)	96 8100 (0.55)	723 200 (0.41)	1 329 600 (0.76)	536 810 (0.31)	935 260 (0.54)	417 550 (0.24)	1 224 020 (0.70)	633 100 (0.36)

Abbreviations: CD, Crohn’s disease; IBD, inflammatory bowel disease; OR, odds ratio (relative to non-IBD control group); OSA, obstructive sleep apnea; UC, ulcerative colitis.

A multivariate analysis with OSA as the dependent variable was conducted in order to adjust for the covariates listed above. The raw data for the analysis can be viewed in the Supplementary Material. Table 3 details the results of the analysis. Age above 65, Caucasian race, male sex, obesity, smoking, hypertension, and diabetes were all independent risk factors for the development of OSA. Obesity represented the most significant risk factor (OR 6.41, 95% CI 6.38–6.44). In addition, after adjusting for the other variables listed, this analysis demonstrated that IBD was independently associated with OSA (OR 1.46, 95% CI 1.43–1.48).

**Table 3.** Multivariate analysis with OSA representing the dependent variable.

	*P*	Odds ratio	95% CI (lower)	95% CI (upper)
Age >65	<.0001	1.524	1.517	1.530
Caucasian	<.0001	1.570	1.563	1.578
Male	<.0001	1.730	1.723	1.737
Obese	<.0001	6.412	6.383	6.440
Smoking	<.0001	1.407	1.400	1.414
Hypertension	<.0001	2.185	2.174	2.196
Diabetes mellitus	<.0001	2.480	2.468	2.491
Inflammatory bowel disease	<.0001	1.459	1.434	1.483

Abbreviation: OSA, obstructive sleep apnea.

## Discussion

Previous studies have demonstrated an association between IBD and extraintestinal inflammation, including persistent airway inflammation.^4^ Inflammation of the upper airways is a known pathophysiologic mechanism for the development of OSA.^1^ However, until this retrospective analysis, no studies had investigated the effects of IBD on the subsequent development of OSA. In this study, we observed that IBD was independently associated with an increased prevalence of OSA.

A statistically significant increase of OSA in patients with underlying IBD was demonstrated in the initial unadjusted analysis. However, several differences in baseline characteristics among the CD, UC, and non-IBD subgroups made interpreting these data difficult. As a result, a multivariate analysis adjusting for major risk factors was conducted. In addition to confirming increased rates of OSA for each known risk factor (age >65, Caucasian race, male sex, obesity, tobacco use, hypertension, and diabetes mellitus), IBD was shown to independently associated with OSA.

This proinflammatory state in IBD is 1 potential mechanism for the development of OSA. This has been previously described in other inflammatory conditions. For example, 1 previous study demonstrated that a larger proportion of rheumatoid arthritis patients screened as “high risk” for sleep apnea, as compared with a control group.^14^ Inflammatory cytokine levels, such as tissue necrosis factor alpha, have been shown to correlate to the severity of OSA.^15^ IBD similarly can lead to increased levels of inflammatory cytokines and systemic inflammation. In turn, this can lead to local enlargement and edema of soft tissue and subsequent obstruction during sleep, resulting in apnea and hypopnea.

Via similar mechanisms, as a result of the proinflammatory environment, IBD has previously been demonstrated to be associated with higher rates of cardiovascular disease, independent of other risk factors. For example, in Panhwar et al, IBD was found to confer a higher risk of acute myocardial infarction with an adjusted OR of 1.25 (95% CI 1.24–1.27, *P* < .0001).^5^ One proposed pathophysiologic mechanism is the chronic inflammation of IBD leading to increasing arterial stiffness and impaired endothelial function.^16^

There are several potential implications of the increased risk of OSA in IBD. For example, it may be beneficial to screen patients with IBD for OSA more frequently than the general population. In addition, more aggressive cardiovascular risk factor management may be indicated in patients with both IBD and OSA given their increased risk of cardiovascular disease. Currently, it is unclear how the severity and treatment of IBD affect the risk of OSA, but this could be addressed in further studies and would further influence screening and management.

Strengths of this study include the large population size due to the use of a database like Explorys. This large dataset allows for more precise results with narrow CIs for each OR for known risk factors of OSA, as well as IBD as an independent risk factor. In addition, the relationship between IBD and OSA is demonstrated in both an unadjusted and adjusted analysis.

The Explorys database has inherent limitations, as well. This includes the fact that patients may seek some of their care at healthcare systems within the Explorys network and some of their care at outside facilities. In addition, Explorys relies on SNOMED-CT diagnosis codes. This may not accurately identify all patients with these conditions and provides no verification these diagnoses are correct. For example, there was no way of confirming the diagnosis of OSA was based on a formal sleep study. Due to the limitations of Explorys, we were also unable to collect data regarding the extent, location, and severity of pathology in those with IBD. These factors, especially severity, may affect the association with OSA. Last, though we were able to correct for obesity using the multivariate analysis, body mass index data for these patients were not available and may be different among the cohorts.

Finally, ascertainment bias is a significant limitation in this study population, as IBD patients may be more involved with the healthcare system than the average patient. This leads to an increased chance of being diagnosed with our studied comorbidities, as well as OSA.

As above, future research could confirm these data prospectively, in addition to clarifying if the risk of OSA increases with increasing severity of IBD, as well as how treatment of IBD affects the risk of developing OSA. Future studies could also investigate outcomes in patients with IBD who develop OSA, as it is currently unknown if OSA and IBD have additive effects in terms of cardiovascular risk. In summary, this is one of the first studies to show that IBD is an independent predictor of OSA. As such, we recommended screening for OSA in patients with IBD, especially in those with 1 or more traditional risk factors for OSA and/or sleep dysregulation.

## Supplementary Material

otac026_suppl_Supplementary_Data

## Data Availability

The data for this article are available throughout its body and in supplementary material.

## Notes

Conference presentation: ACG 2020 (Virtual) (presented in part [univariate analysis only]).

## References

[1] Ryan CM , Bradley TD. Pathogenesis of obstructive sleep apnea. J Appl Physiol. 2005;99(6):2440–2450. 16288102 10.1152/japplphysiol.00772.2005

[2] Arora SS , Malik TA. Inflammatory bowel disease: epidemiology. In: Huber S, ed. New Insights into Inflammatory Bowel Disease. InTech; 2016:1–20.

[3] Hendrickson BA , Gokhale R, Cho JH. Clinical aspects and pathophysiology of inflammatory bowel disease. Clin Microbiol Rev. 2002;15(1):79–94. 11781268 10.1128/CMR.15.1.79-94.2002 PMC118061

[4] Rothfuss KS , Stange EF, Herrlinger KR. Extraintestinal manifestations and complications in inflammatory bowel diseases. World J Gastroenterol. 2006;12(30):4819–4831. 16937463 10.3748/wjg.v12.i30.4819 PMC4087615

[5] Panhwar MS , Mansoor E, Al-Kindi SG, et al. Risk of myocardial infarction in inflammatory bowel disease: a population-based national study. Inflamm Bowel Dis. 2019;25(6):1080–1087. 30500938 10.1093/ibd/izy354

[6] Parish JM , Somers VK. Obstructive sleep apnea and cardiovascular disease. Mayo Clin Proc. 2004;79(8):1036–1046. 15301332 10.4065/79.8.1036

[7] IBM Watson Health. Data sheet. Accessed March 22, 2020. https://www.ibm.com/downloads/cas/6VQK0DLL

[8] Kaelber DC , Foster W, Gilder J, Love TE, Jain AK. Patient characteristics associated with venous thromboembolic events: a cohort study using pooled electronic health record data. J Am Med Inform Assoc. 2012;19(6):965–972. 22759621 10.1136/amiajnl-2011-000782 PMC3534456

[9] El-Assaad I , Al-Kindi SG, Saarel EV, Aziz PF. Lone pediatric atrial fibrillation in the United States: analysis of over 1500 cases. Pediatr Cardiol. 2017;38(5):1004–1009. 28374048 10.1007/s00246-017-1608-7

[10] Mansoor E , Cooper GS. The 2010–2015 prevalence of eosinophilic esophagitis in the USA: a population-based study. Dig Dis Sci. 2016;61(10):2928–2934. 27250980 10.1007/s10620-016-4204-4 PMC5021560

[11] Young T , Skatrud J, Peppard PE. Risk factors for obstructive sleep apnea in adults. JAMA. 2004;291(16):2013–2016. 15113821 10.1001/jama.291.16.2013

[12] Wetter DW , Young TB, Bidwell TR, Badr MS, Palta M. Smoking as a risk factor for sleep-disordered breathing. Arch Intern Med. 1994;154(19):2219–2224. 7944843

[13] Chi L , Comyn FL, Keenan BT, et al. Heritability of craniofacial structures in normal subjects and patients with sleep apnea. Sleep. 2014;37(10):1689–1698. 25197806 10.5665/sleep.4082 PMC4173925

[14] Reading SR , Crowson CS, Rodeheffer RJ, Fitz-Gibbon PD, Maradit-Kremers H, Gabriel SE. Do rheumatoid arthritis patients have a higher risk for sleep apnea? J Rheumatol. 2009;36(9):1869–1872. 19648298 10.3899/jrheum.081335 PMC2834190

[15] Taylor-Gjevre RM , Nair BV, Gjevre JA. Obstructive sleep apnoea in relation to rheumatic disease. Rheumatology. 2013;52(1):15–21. 22923759 10.1093/rheumatology/kes210

[16] Zanoli L , Rastelli S, Inserra G, Castellino P. Arterial structure and function in inflammatory bowel disease. World J Gastroenterol. 2015;21(40):11304–11311. 26523102 10.3748/wjg.v21.i40.11304 PMC4616206

