# Supplementary material for: Prevalence of Obstructive Sleep Apnea Is Increased in Patients With Inflammatory Bowel Disease: A Large, Multi-Network Study
Source: Crohns Colitis 360. 2022 Jul 6;4(3):otac026. doi: 10.1093/crocol/otac026 (PMC9802032; doi:10.1093/crocol/otac026)
Supplement: otac026_suppl_Supplementary_Data [file otac026_suppl_supplementary_data.docx]

| # | Age 65+ | Caucasian | Male | Obesity | Tobacco Use | OSA | HTN | DM | IBD | Frequency (Number of Patients) |
| --- | --- | --- | --- | --- | --- | --- | --- | --- | --- | --- |
| 1 | Y | Y | Y | Y | Y | Y | Y | Y | Y | 170 |
| 2 | Y | Y | Y | Y | Y | Y | Y | Y | N | 7740 |
| 3 | Y | Y | Y | Y | Y | Y | Y | N | Y | 50 |
| 4 | Y | Y | Y | Y | Y | Y | Y | N | N | 3390 |
| 5 | Y | Y | Y | Y | Y | Y | N | Y | Y | 110 |
| 6 | Y | Y | Y | Y | Y | Y | N | Y | N | 6880 |
| 7 | Y | Y | Y | Y | Y | Y | N | N | Y | 60 |
| 8 | Y | Y | Y | Y | Y | Y | N | N | N | 4400 |
| 9 | Y | Y | Y | Y | Y | N | Y | Y | Y | 130 |
| 10 | Y | Y | Y | Y | Y | N | Y | Y | N | 6360 |
| 11 | Y | Y | Y | Y | Y | N | Y | N | Y | 60 |
| 12 | Y | Y | Y | Y | Y | N | Y | N | N | 4460 |
| 13 | Y | Y | Y | Y | Y | N | N | Y | Y | 90 |
| 14 | Y | Y | Y | Y | Y | N | N | Y | N | 7640 |
| 15 | Y | Y | Y | Y | Y | N | N | N | Y | 110 |
| 16 | Y | Y | Y | Y | Y | N | N | N | N | 8190 |
| 17 | Y | Y | Y | Y | N | Y | Y | Y | Y | 350 |
| 18 | Y | Y | Y | Y | N | Y | Y | Y | N | 15070 |
| 19 | Y | Y | Y | Y | N | Y | Y | N | Y | 130 |
| 20 | Y | Y | Y | Y | N | Y | Y | N | N | 8240 |
| 21 | Y | Y | Y | Y | N | Y | N | Y | Y | 200 |
| 22 | Y | Y | Y | Y | N | Y | N | Y | N | 16790 |
| 23 | Y | Y | Y | Y | N | Y | N | N | Y | 200 |
| 24 | Y | Y | Y | Y | N | Y | N | N | N | 13680 |
| 25 | Y | Y | Y | Y | N | N | Y | Y | Y | 280 |
| 26 | Y | Y | Y | Y | N | N | Y | Y | N | 15620 |
| 27 | Y | Y | Y | Y | N | N | Y | N | Y | 240 |
| 28 | Y | Y | Y | Y | N | N | Y | N | N | 13570 |
| 29 | Y | Y | Y | Y | N | N | N | Y | Y | 260 |
| 30 | Y | Y | Y | Y | N | N | N | Y | N | 21900 |
| 31 | Y | Y | Y | Y | N | N | N | N | Y | 360 |
| 32 | Y | Y | Y | Y | N | N | N | N | N | 29740 |
| 33 | Y | Y | Y | N | Y | Y | Y | Y | Y | 160 |
| 34 | Y | Y | Y | N | Y | Y | Y | Y | N | 7320 |
| 35 | Y | Y | Y | N | Y | Y | Y | N | Y | 130 |
| 36 | Y | Y | Y | N | Y | Y | Y | N | N | 6790 |
| 37 | Y | Y | Y | N | Y | Y | N | Y | Y | 150 |
| 38 | Y | Y | Y | N | Y | Y | N | Y | N | 8700 |
| 39 | Y | Y | Y | N | Y | Y | N | N | Y | 160 |
| 40 | Y | Y | Y | N | Y | Y | N | N | N | 13240 |
| 41 | Y | Y | Y | N | Y | N | Y | Y | Y | 370 |
| 42 | Y | Y | Y | N | Y | N | Y | Y | N | 22590 |
| 43 | Y | Y | Y | N | Y | N | Y | N | Y | 490 |
| 44 | Y | Y | Y | N | Y | N | Y | N | N | 34050 |
| 45 | Y | Y | Y | N | Y | N | N | Y | Y | 440 |
| 46 | Y | Y | Y | N | Y | N | N | Y | N | 43500 |
| 47 | Y | Y | Y | N | Y | N | N | N | Y | 1120 |
| 48 | Y | Y | Y | N | Y | N | N | N | N | 122780 |
| 49 | Y | Y | Y | N | N | Y | Y | Y | Y | 360 |
| 50 | Y | Y | Y | N | N | Y | Y | Y | N | 16150 |
| 51 | Y | Y | Y | N | N | Y | Y | N | Y | 350 |
| 52 | Y | Y | Y | N | N | Y | Y | N | N | 18340 |
| 53 | Y | Y | Y | N | N | Y | N | Y | Y | 350 |
| 54 | Y | Y | Y | N | N | Y | N | Y | N | 26670 |
| 55 | Y | Y | Y | N | N | Y | N | N | Y | 590 |
| 56 | Y | Y | Y | N | N | Y | N | N | N | 49540 |
| 57 | Y | Y | Y | N | N | N | Y | Y | Y | 1060 |
| 58 | Y | Y | Y | N | N | N | Y | Y | N | 63630 |
| 59 | Y | Y | Y | N | N | N | Y | N | Y | 1590 |
| 60 | Y | Y | Y | N | N | N | Y | N | N | 107620 |
| 61 | Y | Y | Y | N | N | N | N | Y | Y | 1540 |
| 62 | Y | Y | Y | N | N | N | N | Y | N | 165560 |
| 63 | Y | Y | Y | N | N | N | N | N | Y | 4620 |
| 64 | Y | Y | Y | N | N | N | N | N | N | 619210 |
| 65 | Y | Y | N | Y | Y | Y | Y | Y | Y | 190 |
| 66 | Y | Y | N | Y | Y | Y | Y | Y | N | 5330 |
| 67 | Y | Y | N | Y | Y | Y | Y | N | Y | 70 |
| 68 | Y | Y | N | Y | Y | Y | Y | N | N | 2370 |
| 69 | Y | Y | N | Y | Y | Y | N | Y | Y | 110 |
| 70 | Y | Y | N | Y | Y | Y | N | Y | N | 4520 |
| 71 | Y | Y | N | Y | Y | Y | N | N | Y | 70 |
| 72 | Y | Y | N | Y | Y | Y | N | N | N | 3000 |
| 73 | Y | Y | N | Y | Y | N | Y | Y | Y | 210 |
| 74 | Y | Y | N | Y | Y | N | Y | Y | N | 6560 |
| 75 | Y | Y | N | Y | Y | N | Y | N | Y | 140 |
| 76 | Y | Y | N | Y | Y | N | Y | N | N | 5120 |
| 77 | Y | Y | N | Y | Y | N | N | Y | Y | 150 |
| 78 | Y | Y | N | Y | Y | N | N | Y | N | 7190 |
| 79 | Y | Y | N | Y | Y | N | N | N | Y | 190 |
| 80 | Y | Y | N | Y | Y | N | N | N | N | 9290 |
| 81 | Y | Y | N | Y | N | Y | Y | Y | Y | 530 |
| 82 | Y | Y | N | Y | N | Y | Y | Y | N | 15290 |
| 83 | Y | Y | N | Y | N | Y | Y | N | Y | 250 |
| 84 | Y | Y | N | Y | N | Y | Y | N | N | 8880 |
| 85 | Y | Y | N | Y | N | Y | N | Y | Y | 310 |
| 86 | Y | Y | N | Y | N | Y | N | Y | N | 15370 |
| 87 | Y | Y | N | Y | N | Y | N | N | Y | 250 |
| 88 | Y | Y | N | Y | N | Y | N | N | N | 13840 |
| 89 | Y | Y | N | Y | N | N | Y | Y | Y | 620 |
| 90 | Y | Y | N | Y | N | N | Y | Y | N | 25800 |
| 91 | Y | Y | N | Y | N | N | Y | N | Y | 560 |
| 92 | Y | Y | N | Y | N | N | Y | N | N | 26450 |
| 93 | Y | Y | N | Y | N | N | N | Y | Y | 520 |
| 94 | Y | Y | N | Y | N | N | N | Y | N | 33280 |
| 95 | Y | Y | N | Y | N | N | N | N | Y | 820 |
| 96 | Y | Y | N | Y | N | N | N | N | N | 54210 |
| 97 | Y | Y | N | N | Y | Y | Y | Y | Y | 140 |
| 98 | Y | Y | N | N | Y | Y | Y | Y | N | 3540 |
| 99 | Y | Y | N | N | Y | Y | Y | N | Y | 150 |
| 100 | Y | Y | N | N | Y | Y | Y | N | N | 3860 |
| 101 | Y | Y | N | N | Y | Y | N | Y | Y | 100 |
| 102 | Y | Y | N | N | Y | Y | N | Y | N | 4320 |
| 103 | Y | Y | N | N | Y | Y | N | N | Y | 170 |
| 104 | Y | Y | N | N | Y | Y | N | N | N | 7970 |
| 105 | Y | Y | N | N | Y | N | Y | Y | Y | 520 |
| 106 | Y | Y | N | N | Y | N | Y | Y | N | 18470 |
| 107 | Y | Y | N | N | Y | N | Y | N | Y | 910 |
| 108 | Y | Y | N | N | Y | N | Y | N | N | 34740 |
| 109 | Y | Y | N | N | Y | N | N | Y | Y | 540 |
| 110 | Y | Y | N | N | Y | N | N | Y | N | 32570 |
| 111 | Y | Y | N | N | Y | N | N | N | Y | 1740 |
| 112 | Y | Y | N | N | Y | N | N | N | N | 122580 |
| 113 | Y | Y | N | N | N | Y | Y | Y | Y | 330 |
| 114 | Y | Y | N | N | N | Y | Y | Y | N | 11210 |
| 115 | Y | Y | N | N | N | Y | Y | N | Y | 390 |
| 116 | Y | Y | N | N | N | Y | Y | N | N | 14200 |
| 117 | Y | Y | N | N | N | Y | N | Y | Y | 280 |
| 118 | Y | Y | N | N | N | Y | N | Y | N | 16950 |
| 119 | Y | Y | N | N | N | Y | N | N | Y | 600 |
| 120 | Y | Y | N | N | N | Y | N | N | N | 36380 |
| 121 | Y | Y | N | N | N | N | Y | Y | Y | 1720 |
| 122 | Y | Y | N | N | N | N | Y | Y | N | 76620 |
| 123 | Y | Y | N | N | N | N | Y | N | Y | 3530 |
| 124 | Y | Y | N | N | N | N | Y | N | N | 168540 |
| 125 | Y | Y | N | N | N | N | N | Y | Y | 2020 |
| 126 | Y | Y | N | N | N | N | N | Y | N | 178460 |
| 127 | Y | Y | N | N | N | N | N | N | Y | 8450 |
| 128 | Y | Y | N | N | N | N | N | N | N | 910880 |
| 129 | Y | N | Y | Y | Y | Y | Y | Y | Y | 10 |
| 130 | Y | N | Y | Y | Y | Y | Y | Y | N | 910 |
| 131 | Y | N | Y | Y | Y | Y | Y | N | Y | 5 |
| 132 | Y | N | Y | Y | Y | Y | Y | N | N | 230 |
| 133 | Y | N | Y | Y | Y | Y | N | Y | Y | 5 |
| 134 | Y | N | Y | Y | Y | Y | N | Y | N | 850 |
| 135 | Y | N | Y | Y | Y | Y | N | N | Y | 5 |
| 136 | Y | N | Y | Y | Y | Y | N | N | N | 360 |
| 137 | Y | N | Y | Y | Y | N | Y | Y | Y | 5 |
| 138 | Y | N | Y | Y | Y | N | Y | Y | N | 800 |
| 139 | Y | N | Y | Y | Y | N | Y | N | Y | 5 |
| 140 | Y | N | Y | Y | Y | N | Y | N | N | 410 |
| 141 | Y | N | Y | Y | Y | N | N | Y | Y | 10 |
| 142 | Y | N | Y | Y | Y | N | N | Y | N | 1300 |
| 143 | Y | N | Y | Y | Y | N | N | N | Y | 5 |
| 144 | Y | N | Y | Y | Y | N | N | N | N | 960 |
| 145 | Y | N | Y | Y | N | Y | Y | Y | Y | 40 |
| 146 | Y | N | Y | Y | N | Y | Y | Y | N | 2430 |
| 147 | Y | N | Y | Y | N | Y | Y | N | Y | 5 |
| 148 | Y | N | Y | Y | N | Y | Y | N | N | 890 |
| 149 | Y | N | Y | Y | N | Y | N | Y | Y | 30 |
| 150 | Y | N | Y | Y | N | Y | N | Y | N | 3390 |
| 151 | Y | N | Y | Y | N | Y | N | N | Y | 10 |
| 152 | Y | N | Y | Y | N | Y | N | N | N | 1980 |
| 153 | Y | N | Y | Y | N | N | Y | Y | Y | 40 |
| 154 | Y | N | Y | Y | N | N | Y | Y | N | 2950 |
| 155 | Y | N | Y | Y | N | N | Y | N | Y | 20 |
| 156 | Y | N | Y | Y | N | N | Y | N | N | 1720 |
| 157 | Y | N | Y | Y | N | N | N | Y | Y | 50 |
| 158 | Y | N | Y | Y | N | N | N | Y | N | 5750 |
| 159 | Y | N | Y | Y | N | N | N | N | Y | 40 |
| 160 | Y | N | Y | Y | N | N | N | N | N | 5960 |
| 161 | Y | N | Y | N | Y | Y | Y | Y | Y | 30 |
| 162 | Y | N | Y | N | Y | Y | Y | Y | N | 980 |
| 163 | Y | N | Y | N | Y | Y | Y | N | Y | 5 |
| 164 | Y | N | Y | N | Y | Y | Y | N | N | 610 |
| 165 | Y | N | Y | N | Y | Y | N | Y | Y | 5 |
| 166 | Y | N | Y | N | Y | Y | N | Y | N | 1510 |
| 167 | Y | N | Y | N | Y | Y | N | N | Y | 10 |
| 168 | Y | N | Y | N | Y | Y | N | N | N | 1470 |
| 169 | Y | N | Y | N | Y | N | Y | Y | Y | 50 |
| 170 | Y | N | Y | N | Y | N | Y | Y | N | 4560 |
| 171 | Y | N | Y | N | Y | N | Y | N | Y | 50 |
| 172 | Y | N | Y | N | Y | N | Y | N | N | 4820 |
| 173 | Y | N | Y | N | Y | N | N | Y | Y | 80 |
| 174 | Y | N | Y | N | Y | N | N | Y | N | 11710 |
| 175 | Y | N | Y | N | Y | N | N | N | Y | 90 |
| 176 | Y | N | Y | N | Y | N | N | N | N | 26110 |
| 177 | Y | N | Y | N | N | Y | Y | Y | Y | 60 |
| 178 | Y | N | Y | N | N | Y | Y | Y | N | 3100 |
| 179 | Y | N | Y | N | N | Y | Y | N | Y | 20 |
| 180 | Y | N | Y | N | N | Y | Y | N | N | 2560 |
| 181 | Y | N | Y | N | N | Y | N | Y | Y | 60 |
| 182 | Y | N | Y | N | N | Y | N | Y | N | 6350 |
| 183 | Y | N | Y | N | N | Y | N | N | Y | 70 |
| 184 | Y | N | Y | N | N | Y | N | N | N | 10240 |
| 185 | Y | N | Y | N | N | N | Y | Y | Y | 160 |
| 186 | Y | N | Y | N | N | N | Y | Y | N | 16500 |
| 187 | Y | N | Y | N | N | N | Y | N | Y | 170 |
| 188 | Y | N | Y | N | N | N | Y | N | N | 18780 |
| 189 | Y | N | Y | N | N | N | N | Y | Y | 320 |
| 190 | Y | N | Y | N | N | N | N | Y | N | 62650 |
| 191 | Y | N | Y | N | N | N | N | N | Y | 690 |
| 192 | Y | N | Y | N | N | N | N | N | N | 287020 |
| 193 | Y | N | N | Y | Y | Y | Y | Y | Y | 40 |
| 194 | Y | N | N | Y | Y | Y | Y | Y | N | 1210 |
| 195 | Y | N | N | Y | Y | Y | Y | N | Y | 10 |
| 196 | Y | N | N | Y | Y | Y | Y | N | N | 370 |
| 197 | Y | N | N | Y | Y | Y | N | Y | Y | 20 |
| 198 | Y | N | N | Y | Y | Y | N | Y | N | 1020 |
| 199 | Y | N | N | Y | Y | Y | N | N | Y | 10 |
| 200 | Y | N | N | Y | Y | Y | N | N | N | 440 |
| 201 | Y | N | N | Y | Y | N | Y | Y | Y | 40 |
| 202 | Y | N | N | Y | Y | N | Y | Y | N | 1450 |
| 203 | Y | N | N | Y | Y | N | Y | N | Y | 20 |
| 204 | Y | N | N | Y | Y | N | Y | N | N | 820 |
| 205 | Y | N | N | Y | Y | N | N | Y | Y | 20 |
| 206 | Y | N | N | Y | Y | N | N | Y | N | 1750 |
| 207 | Y | N | N | Y | Y | N | N | N | Y | 10 |
| 208 | Y | N | N | Y | Y | N | N | N | N | 1570 |
| 209 | Y | N | N | Y | N | Y | Y | Y | Y | 140 |
| 210 | Y | N | N | Y | N | Y | Y | Y | N | 5200 |
| 211 | Y | N | N | Y | N | Y | Y | N | Y | 40 |
| 212 | Y | N | N | Y | N | Y | Y | N | N | 1960 |
| 213 | Y | N | N | Y | N | Y | N | Y | Y | 90 |
| 214 | Y | N | N | Y | N | Y | N | Y | N | 5590 |
| 215 | Y | N | N | Y | N | Y | N | N | Y | 40 |
| 216 | Y | N | N | Y | N | Y | N | N | N | 3570 |
| 217 | Y | N | N | Y | N | N | Y | Y | Y | 140 |
| 218 | Y | N | N | Y | N | N | Y | Y | N | 7930 |
| 219 | Y | N | N | Y | N | N | Y | N | Y | 90 |
| 220 | Y | N | N | Y | N | N | Y | N | N | 5280 |
| 221 | Y | N | N | Y | N | N | N | Y | Y | 90 |
| 222 | Y | N | N | Y | N | N | N | Y | N | 13480 |
| 223 | Y | N | N | Y | N | N | N | N | Y | 120 |
| 224 | Y | N | N | Y | N | N | N | N | N | 15510 |
| 225 | Y | N | N | N | Y | Y | Y | Y | Y | 30 |
| 226 | Y | N | N | N | Y | Y | Y | Y | N | 820 |
| 227 | Y | N | N | N | Y | Y | Y | N | Y | 5 |
| 228 | Y | N | N | N | Y | Y | Y | N | N | 440 |
| 229 | Y | N | N | N | Y | Y | N | Y | Y | 20 |
| 230 | Y | N | N | N | Y | Y | N | Y | N | 1000 |
| 231 | Y | N | N | N | Y | Y | N | N | Y | 10 |
| 232 | Y | N | N | N | Y | Y | N | N | N | 1030 |
| 233 | Y | N | N | N | Y | N | Y | Y | Y | 90 |
| 234 | Y | N | N | N | Y | N | Y | Y | N | 4380 |
| 235 | Y | N | N | N | Y | N | Y | N | Y | 60 |
| 236 | Y | N | N | N | Y | N | Y | N | N | 4560 |
| 237 | Y | N | N | N | Y | N | N | Y | Y | 70 |
| 238 | Y | N | N | N | Y | N | N | Y | N | 9470 |
| 239 | Y | N | N | N | Y | N | N | N | Y | 170 |
| 240 | Y | N | N | N | Y | N | N | N | N | 20660 |
| 241 | Y | N | N | N | N | Y | Y | Y | Y | 90 |
| 242 | Y | N | N | N | N | Y | Y | Y | N | 3410 |
| 243 | Y | N | N | N | N | Y | Y | N | Y | 50 |
| 244 | Y | N | N | N | N | Y | Y | N | N | 2500 |
| 245 | Y | N | N | N | N | Y | N | Y | Y | 60 |
| 246 | Y | N | N | N | N | Y | N | Y | N | 6150 |
| 247 | Y | N | N | N | N | Y | N | N | Y | 100 |
| 248 | Y | N | N | N | N | Y | N | N | N | 11370 |
| 249 | Y | N | N | N | N | N | Y | Y | Y | 390 |
| 250 | Y | N | N | N | N | N | Y | Y | N | 25430 |
| 251 | Y | N | N | N | N | N | Y | N | Y | 380 |
| 252 | Y | N | N | N | N | N | Y | N | N | 31280 |
| 253 | Y | N | N | N | N | N | N | Y | Y | 420 |
| 254 | Y | N | N | N | N | N | N | Y | N | 86380 |
| 255 | Y | N | N | N | N | N | N | N | Y | 1300 |
| 256 | Y | N | N | N | N | N | N | N | N | 446450 |
| 257 | N | Y | Y | Y | Y | Y | Y | Y | Y | 160 |
| 258 | N | Y | Y | Y | Y | Y | Y | Y | N | 7040 |
| 259 | N | Y | Y | Y | Y | Y | Y | N | Y | 70 |
| 260 | N | Y | Y | Y | Y | Y | Y | N | N | 4740 |
| 261 | N | Y | Y | Y | Y | Y | N | Y | Y | 170 |
| 262 | N | Y | Y | Y | Y | Y | N | Y | N | 10550 |
| 263 | N | Y | Y | Y | Y | Y | N | N | Y | 210 |
| 264 | N | Y | Y | Y | Y | Y | N | N | N | 14540 |
| 265 | N | Y | Y | Y | Y | N | Y | Y | Y | 110 |
| 266 | N | Y | Y | Y | Y | N | Y | Y | N | 5390 |
| 267 | N | Y | Y | Y | Y | N | Y | N | Y | 120 |
| 268 | N | Y | Y | Y | Y | N | Y | N | N | 7000 |
| 269 | N | Y | Y | Y | Y | N | N | Y | Y | 160 |
| 270 | N | Y | Y | Y | Y | N | N | Y | N | 13290 |
| 271 | N | Y | Y | Y | Y | N | N | N | Y | 520 |
| 272 | N | Y | Y | Y | Y | N | N | N | N | 39470 |
| 273 | N | Y | Y | Y | N | Y | Y | Y | Y | 200 |
| 274 | N | Y | Y | Y | N | Y | Y | Y | N | 10110 |
| 275 | N | Y | Y | Y | N | Y | Y | N | Y | 140 |
| 276 | N | Y | Y | Y | N | Y | Y | N | N | 9050 |
| 277 | N | Y | Y | Y | N | Y | N | Y | Y | 250 |
| 278 | N | Y | Y | Y | N | Y | N | Y | N | 16530 |
| 279 | N | Y | Y | Y | N | Y | N | N | Y | 420 |
| 280 | N | Y | Y | Y | N | Y | N | N | N | 30860 |
| 281 | N | Y | Y | Y | N | N | Y | Y | Y | 140 |
| 282 | N | Y | Y | Y | N | N | Y | Y | N | 9280 |
| 283 | N | Y | Y | Y | N | N | Y | N | Y | 180 |
| 284 | N | Y | Y | Y | N | N | Y | N | N | 14530 |
| 285 | N | Y | Y | Y | N | N | N | Y | Y | 230 |
| 286 | N | Y | Y | Y | N | N | N | Y | N | 23550 |
| 287 | N | Y | Y | Y | N | N | N | N | Y | 1010 |
| 288 | N | Y | Y | Y | N | N | N | N | N | 89550 |
| 289 | N | Y | Y | N | Y | Y | Y | Y | Y | 100 |
| 290 | N | Y | Y | N | Y | Y | Y | Y | N | 4650 |
| 291 | N | Y | Y | N | Y | Y | Y | N | Y | 120 |
| 292 | N | Y | Y | N | Y | Y | Y | N | N | 6820 |
| 293 | N | Y | Y | N | Y | Y | N | Y | Y | 140 |
| 294 | N | Y | Y | N | Y | Y | N | Y | N | 10380 |
| 295 | N | Y | Y | N | Y | Y | N | N | Y | 480 |
| 296 | N | Y | Y | N | Y | Y | N | N | N | 36130 |
| 297 | N | Y | Y | N | Y | N | Y | Y | Y | 230 |
| 298 | N | Y | Y | N | Y | N | Y | Y | N | 13910 |
| 299 | N | Y | Y | N | Y | N | Y | N | Y | 480 |
| 300 | N | Y | Y | N | Y | N | Y | N | N | 36490 |
| 301 | N | Y | Y | N | Y | N | N | Y | Y | 580 |
| 302 | N | Y | Y | N | Y | N | N | Y | N | 69360 |
| 303 | N | Y | Y | N | Y | N | N | N | Y | 5490 |
| 304 | N | Y | Y | N | Y | N | N | N | N | 707330 |
| 305 | N | Y | Y | N | N | Y | Y | Y | Y | 130 |
| 306 | N | Y | Y | N | N | Y | Y | Y | N | 7210 |
| 307 | N | Y | Y | N | N | Y | Y | N | Y | 200 |
| 308 | N | Y | Y | N | N | Y | Y | N | N | 13150 |
| 309 | N | Y | Y | N | N | Y | N | Y | Y | 210 |
| 310 | N | Y | Y | N | N | Y | N | Y | N | 18600 |
| 311 | N | Y | Y | N | N | Y | N | N | Y | 1060 |
| 312 | N | Y | Y | N | N | Y | N | N | N | 92550 |
| 313 | N | Y | Y | N | N | N | Y | Y | Y | 320 |
| 314 | N | Y | Y | N | N | N | Y | Y | N | 22910 |
| 315 | N | Y | Y | N | N | N | Y | N | Y | 870 |
| 316 | N | Y | Y | N | N | N | Y | N | N | 66530 |
| 317 | N | Y | Y | N | N | N | N | Y | Y | 1060 |
| 318 | N | Y | Y | N | N | N | N | Y | N | 129760 |
| 319 | N | Y | Y | N | N | N | N | N | Y | 15640 |
| 320 | N | Y | Y | N | N | N | N | N | N | 2563500 |
| 321 | N | Y | N | Y | Y | Y | Y | Y | Y | 250 |
| 322 | N | Y | N | Y | Y | Y | Y | Y | N | 5940 |
| 323 | N | Y | N | Y | Y | Y | Y | N | Y | 100 |
| 324 | N | Y | N | Y | Y | Y | Y | N | N | 3340 |
| 325 | N | Y | N | Y | Y | Y | N | Y | Y | 290 |
| 326 | N | Y | N | Y | Y | Y | N | Y | N | 10720 |
| 327 | N | Y | N | Y | Y | Y | N | N | Y | 270 |
| 328 | N | Y | N | Y | Y | Y | N | N | N | 13330 |
| 329 | N | Y | N | Y | Y | N | Y | Y | Y | 210 |
| 330 | N | Y | N | Y | Y | N | Y | Y | N | 6270 |
| 331 | N | Y | N | Y | Y | N | Y | N | Y | 190 |
| 332 | N | Y | N | Y | Y | N | Y | N | N | 7430 |
| 333 | N | Y | N | Y | Y | N | N | Y | Y | 620 |
| 334 | N | Y | N | Y | Y | N | N | Y | N | 22110 |
| 335 | N | Y | N | Y | Y | N | N | N | Y | 1270 |
| 336 | N | Y | N | Y | Y | N | N | N | N | 70260 |
| 337 | N | Y | N | Y | N | Y | Y | Y | Y | 310 |
| 338 | N | Y | N | Y | N | Y | Y | Y | N | 9240 |
| 339 | N | Y | N | Y | N | Y | Y | N | Y | 200 |
| 340 | N | Y | N | Y | N | Y | Y | N | N | 7300 |
| 341 | N | Y | N | Y | N | Y | N | Y | Y | 380 |
| 342 | N | Y | N | Y | N | Y | N | Y | N | 17620 |
| 343 | N | Y | N | Y | N | Y | N | N | Y | 520 |
| 344 | N | Y | N | Y | N | Y | N | N | N | 30190 |
| 345 | N | Y | N | Y | N | N | Y | Y | Y | 300 |
| 346 | N | Y | N | Y | N | N | Y | Y | N | 13320 |
| 347 | N | Y | N | Y | N | N | Y | N | Y | 380 |
| 348 | N | Y | N | Y | N | N | Y | N | N | 20430 |
| 349 | N | Y | N | Y | N | N | N | Y | Y | 1030 |
| 350 | N | Y | N | Y | N | N | N | Y | N | 45590 |
| 351 | N | Y | N | Y | N | N | N | N | Y | 2240 |
| 352 | N | Y | N | Y | N | N | N | N | N | 191400 |
| 353 | N | Y | N | N | Y | Y | Y | Y | Y | 100 |
| 354 | N | Y | N | N | Y | Y | Y | Y | N | 2690 |
| 355 | N | Y | N | N | Y | Y | Y | N | Y | 110 |
| 356 | N | Y | N | N | Y | Y | Y | N | N | 3360 |
| 357 | N | Y | N | N | Y | Y | N | Y | Y | 150 |
| 358 | N | Y | N | N | Y | Y | N | Y | N | 7520 |
| 359 | N | Y | N | N | Y | Y | N | N | Y | 390 |
| 360 | N | Y | N | N | Y | Y | N | N | N | 22950 |
| 361 | N | Y | N | N | Y | N | Y | Y | Y | 290 |
| 362 | N | Y | N | N | Y | N | Y | Y | N | 10850 |
| 363 | N | Y | N | N | Y | N | Y | N | Y | 650 |
| 364 | N | Y | N | N | Y | N | Y | N | N | 29300 |
| 365 | N | Y | N | N | Y | N | N | Y | Y | 2540 |
| 366 | N | Y | N | N | Y | N | N | Y | N | 74370 |
| 367 | N | Y | N | N | Y | N | N | N | Y | 7560 |
| 368 | N | Y | N | N | Y | N | N | N | N | 728900 |
| 369 | N | Y | N | N | N | Y | Y | Y | Y | 120 |
| 370 | N | Y | N | N | N | Y | Y | Y | N | 4470 |
| 371 | N | Y | N | N | N | Y | Y | N | Y | 130 |
| 372 | N | Y | N | N | N | Y | Y | N | N | 6660 |
| 373 | N | Y | N | N | N | Y | N | Y | Y | 220 |
| 374 | N | Y | N | N | N | Y | N | Y | N | 12980 |
| 375 | N | Y | N | N | N | Y | N | N | Y | 860 |
| 376 | N | Y | N | N | N | Y | N | N | N | 59900 |
| 377 | N | Y | N | N | N | N | Y | Y | Y | 390 |
| 378 | N | Y | N | N | N | N | Y | Y | N | 22270 |
| 379 | N | Y | N | N | N | N | Y | N | Y | 1070 |
| 380 | N | Y | N | N | N | N | Y | N | N | 69290 |
| 381 | N | Y | N | N | N | N | N | Y | Y | 5310 |
| 382 | N | Y | N | N | N | N | N | Y | N | 173390 |
| 383 | N | Y | N | N | N | N | N | N | Y | 22170 |
| 384 | N | Y | N | N | N | N | N | N | N | 3438160 |
| 385 | N | N | Y | Y | Y | Y | Y | Y | Y | 30 |
| 386 | N | N | Y | Y | Y | Y | Y | Y | N | 1950 |
| 387 | N | N | Y | Y | Y | Y | Y | N | Y | 20 |
| 388 | N | N | Y | Y | Y | Y | Y | N | N | 950 |
| 389 | N | N | Y | Y | Y | Y | N | Y | Y | 30 |
| 390 | N | N | Y | Y | Y | Y | N | Y | N | 3240 |
| 391 | N | N | Y | Y | Y | Y | N | N | Y | 30 |
| 392 | N | N | Y | Y | Y | Y | N | N | N | 3540 |
| 393 | N | N | Y | Y | Y | N | Y | Y | Y | 20 |
| 394 | N | N | Y | Y | Y | N | Y | Y | N | 1630 |
| 395 | N | N | Y | Y | Y | N | Y | N | Y | 20 |
| 396 | N | N | Y | Y | Y | N | Y | N | N | 1480 |
| 397 | N | N | Y | Y | Y | N | N | Y | Y | 30 |
| 398 | N | N | Y | Y | Y | N | N | Y | N | 4830 |
| 399 | N | N | Y | Y | Y | N | N | N | Y | 110 |
| 400 | N | N | Y | Y | Y | N | N | N | N | 11850 |
| 401 | N | N | Y | Y | N | Y | Y | Y | Y | 70 |
| 402 | N | N | Y | Y | N | Y | Y | Y | N | 3470 |
| 403 | N | N | Y | Y | N | Y | Y | N | Y | 40 |
| 404 | N | N | Y | Y | N | Y | Y | N | N | 2220 |
| 405 | N | N | Y | Y | N | Y | N | Y | Y | 60 |
| 406 | N | N | Y | Y | N | Y | N | Y | N | 6970 |
| 407 | N | N | Y | Y | N | Y | N | N | Y | 80 |
| 408 | N | N | Y | Y | N | Y | N | N | N | 9830 |
| 409 | N | N | Y | Y | N | N | Y | Y | Y | 40 |
| 410 | N | N | Y | Y | N | N | Y | Y | N | 3300 |
| 411 | N | N | Y | Y | N | N | Y | N | Y | 30 |
| 412 | N | N | Y | Y | N | N | Y | N | N | 3700 |
| 413 | N | N | Y | Y | N | N | N | Y | Y | 100 |
| 414 | N | N | Y | Y | N | N | N | Y | N | 12270 |
| 415 | N | N | Y | Y | N | N | N | N | Y | 250 |
| 416 | N | N | Y | Y | N | N | N | N | N | 39840 |
| 417 | N | N | Y | N | Y | Y | Y | Y | Y | 20 |
| 418 | N | N | Y | N | Y | Y | Y | Y | N | 1230 |
| 419 | N | N | Y | N | Y | Y | Y | N | Y | 30 |
| 420 | N | N | Y | N | Y | Y | Y | N | N | 1180 |
| 421 | N | N | Y | N | Y | Y | N | Y | Y | 30 |
| 422 | N | N | Y | N | Y | Y | N | Y | N | 2980 |
| 423 | N | N | Y | N | Y | Y | N | N | Y | 70 |
| 424 | N | N | Y | N | Y | Y | N | N | N | 7880 |
| 425 | N | N | Y | N | Y | N | Y | Y | Y | 80 |
| 426 | N | N | Y | N | Y | N | Y | Y | N | 4800 |
| 427 | N | N | Y | N | Y | N | Y | N | Y | 90 |
| 428 | N | N | Y | N | Y | N | Y | N | N | 8590 |
| 429 | N | N | Y | N | Y | N | N | Y | Y | 170 |
| 430 | N | N | Y | N | Y | N | N | Y | N | 28580 |
| 431 | N | N | Y | N | Y | N | N | N | Y | 1180 |
| 432 | N | N | Y | N | Y | N | N | N | N | 256000 |
| 433 | N | N | Y | N | N | Y | Y | Y | Y | 30 |
| 434 | N | N | Y | N | N | Y | Y | Y | N | 2420 |
| 435 | N | N | Y | N | N | Y | Y | N | Y | 30 |
| 436 | N | N | Y | N | N | Y | Y | N | N | 3120 |
| 437 | N | N | Y | N | N | Y | N | Y | Y | 50 |
| 438 | N | N | Y | N | N | Y | N | Y | N | 7130 |
| 439 | N | N | Y | N | N | Y | N | N | Y | 390 |
| 440 | N | N | Y | N | N | Y | N | N | N | 43250 |
| 441 | N | N | Y | N | N | N | Y | Y | Y | 100 |
| 442 | N | N | Y | N | N | N | Y | Y | N | 10130 |
| 443 | N | N | Y | N | N | N | Y | N | Y | 150 |
| 444 | N | N | Y | N | N | N | Y | N | N | 19700 |
| 445 | N | N | Y | N | N | N | N | Y | Y | 340 |
| 446 | N | N | Y | N | N | N | N | Y | N | 78850 |
| 447 | N | N | Y | N | N | N | N | N | Y | 9010 |
| 448 | N | N | Y | N | N | N | N | N | N | 2774990 |
| 449 | N | N | N | Y | Y | Y | Y | Y | Y | 100 |
| 450 | N | N | N | Y | Y | Y | Y | Y | N | 2720 |
| 451 | N | N | N | Y | Y | Y | Y | N | Y | 30 |
| 452 | N | N | N | Y | Y | Y | Y | N | N | 1370 |
| 453 | N | N | N | Y | Y | Y | N | Y | Y | 80 |
| 454 | N | N | N | Y | Y | Y | N | Y | N | 4250 |
| 455 | N | N | N | Y | Y | Y | N | N | Y | 80 |
| 456 | N | N | N | Y | Y | Y | N | N | N | 4280 |
| 457 | N | N | N | Y | Y | N | Y | Y | Y | 70 |
| 458 | N | N | N | Y | Y | N | Y | Y | N | 2720 |
| 459 | N | N | N | Y | Y | N | Y | N | Y | 40 |
| 460 | N | N | N | Y | Y | N | Y | N | N | 2900 |
| 461 | N | N | N | Y | Y | N | N | Y | Y | 180 |
| 462 | N | N | N | Y | Y | N | N | Y | N | 8700 |
| 463 | N | N | N | Y | Y | N | N | N | Y | 290 |
| 464 | N | N | N | Y | Y | N | N | N | N | 25370 |
| 465 | N | N | N | Y | N | Y | Y | Y | Y | 160 |
| 466 | N | N | N | Y | N | Y | Y | Y | N | 5610 |
| 467 | N | N | N | Y | N | Y | Y | N | Y | 80 |
| 468 | N | N | N | Y | N | Y | Y | N | N | 3640 |
| 469 | N | N | N | Y | N | Y | N | Y | Y | 130 |
| 470 | N | N | N | Y | N | Y | N | Y | N | 10960 |
| 471 | N | N | N | Y | N | Y | N | N | Y | 160 |
| 472 | N | N | N | Y | N | Y | N | N | N | 15690 |
| 473 | N | N | N | Y | N | N | Y | Y | Y | 120 |
| 474 | N | N | N | Y | N | N | Y | Y | N | 7420 |
| 475 | N | N | N | Y | N | N | Y | N | Y | 110 |
| 476 | N | N | N | Y | N | N | Y | N | N | 9490 |
| 477 | N | N | N | Y | N | N | N | Y | Y | 300 |
| 478 | N | N | N | Y | N | N | N | Y | N | 29980 |
| 479 | N | N | N | Y | N | N | N | N | Y | 830 |
| 480 | N | N | N | Y | N | N | N | N | N | 116700 |
| 481 | N | N | N | N | Y | Y | Y | Y | Y | 30 |
| 482 | N | N | N | N | Y | Y | Y | Y | N | 960 |
| 483 | N | N | N | N | Y | Y | Y | N | Y | 20 |
| 484 | N | N | N | N | Y | Y | Y | N | N | 820 |
| 485 | N | N | N | N | Y | Y | N | Y | Y | 30 |
| 486 | N | N | N | N | Y | Y | N | Y | N | 2240 |
| 487 | N | N | N | N | Y | Y | N | N | Y | 50 |
| 488 | N | N | N | N | Y | Y | N | N | N | 4810 |
| 489 | N | N | N | N | Y | N | Y | Y | Y | 100 |
| 490 | N | N | N | N | Y | N | Y | Y | N | 4350 |
| 491 | N | N | N | N | Y | N | Y | N | Y | 120 |
| 492 | N | N | N | N | Y | N | Y | N | N | 7900 |
| 493 | N | N | N | N | Y | N | N | Y | Y | 480 |
| 494 | N | N | N | N | Y | N | N | Y | N | 26990 |
| 495 | N | N | N | N | Y | N | N | N | Y | 1270 |
| 496 | N | N | N | N | Y | N | N | N | N | 206200 |
| 497 | N | N | N | N | N | Y | Y | Y | Y | 40 |
| 498 | N | N | N | N | N | Y | Y | Y | N | 2130 |
| 499 | N | N | N | N | N | Y | Y | N | Y | 40 |
| 500 | N | N | N | N | N | Y | Y | N | N | 2420 |
| 501 | N | N | N | N | N | Y | N | Y | Y | 80 |
| 502 | N | N | N | N | N | Y | N | Y | N | 7040 |
| 503 | N | N | N | N | N | Y | N | N | Y | 360 |
| 504 | N | N | N | N | N | Y | N | N | N | 36760 |
| 505 | N | N | N | N | N | N | Y | Y | Y | 140 |
| 506 | N | N | N | N | N | N | Y | Y | N | 12880 |
| 507 | N | N | N | N | N | N | Y | N | Y | 270 |
| 508 | N | N | N | N | N | N | Y | N | N | 26790 |
| 509 | N | N | N | N | N | N | N | Y | Y | 1720 |
| 510 | N | N | N | N | N | N | N | Y | N | 117170 |
| 511 | N | N | N | N | N | N | N | N | Y | 10690 |
| 512 | N | N | N | N | N | N | N | N | N | 3513040 |

Abbreviations:

Y: Yes

N: No

OSA: obstructive sleep apnea

HTN: hypertension

DM: diabetes mellitus

IBD: inflammatory bowel disease
